# A Survey of Henipavirus Tropism—Our Current Understanding from a Species/Organ and Cellular Level

**DOI:** 10.3390/v15102048

**Published:** 2023-10-04

**Authors:** Sandra Diederich, Shawn Babiuk, Hani Boshra

**Affiliations:** 1 Institute of Novel and Emerging Infectious Diseases, Friedrich-Loeffler-Institut, Federal Research Institute for Animal Health, 17493 Greifswald, Germany; sandra.diederich@fli.de; 2 Canadian Food Inspection Agency, National Centre for Foreign Animal Disease, Winnipeg, MB R3E EM4, Canada; shawn.babiuk@inspection.gc.ca; 3 Global Urgent and Advanced Research and Development (GUARD), 911 rue Principale, Batiscan, QC G0X 1A0, Canada

**Keywords:** henipavirus, RNA virus, tropism, vector transmission, pathology

## Abstract

Henipaviruses are single-stranded RNA viruses that have been shown to be virulent in several species, including humans, pigs, horses, and rodents. Isolated nearly 30 years ago, these viruses have been shown to be of particular concern to public health, as at least two members (Nipah and Hendra viruses) are highly virulent, as well as zoonotic, and are thus classified as BSL4 pathogens. Although only 5 members of this genus have been isolated and characterized, metagenomics analysis using animal fluids and tissues has demonstrated the existence of other novel henipaviruses, suggesting a far greater degree of phylogenetic diversity than is currently known. Using a variety of molecular biology techniques, it has been shown that these viruses exhibit varying degrees of tropism on a species, organ/tissue, and cellular level. This review will attempt to provide a general overview of our current understanding of henipaviruses, with a particular emphasis on viral tropism.

## 1. Introduction

### 1.1. The Genus Henipavirus

Henipaviruses belong to the genus *Henipavirus* within the *Paramyxoviridae* family (International Committee on Taxonomy of Viruses, ICTV); and as of the writing of this manuscript, ICTV currently lists only 5 members of this genus (Hendra, Nipah, Mojiang, Cedar and Ghana virus). However, the recent widespread use of metagenomics and high throughput sequencing has led researchers to discover other novel species of henipaviruses, which will be discussed later in this review. The first member of this family to be discovered was Hendra virus (HeV), following an outbreak among horses and humans in Australia [1,2,3]. Since then, several other members of this genus have been identified in a variety of animals; and two members (Nipah virusNiV, and HeV) have demonstrated a zoonotic potential and have caused mortalities in humans. The degree of tropism, from a species, tissue/organ and cellular level, will be the primary focus of this review.

### 1.2. Genomic Structure

**Figure 1:**
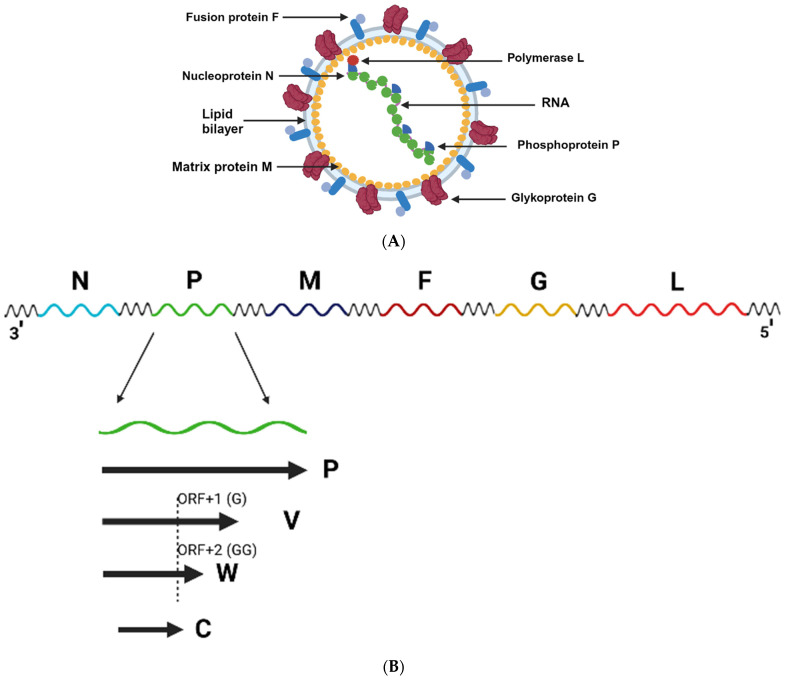
Structural and genomic representation of a prototypical henipavirus. (**A**) A prototypical henipavirus virion is composed of 6 major structural proteins: nucleocapsid protein (N), phosphoprotein (P), matrix protein (M), fusion protein (F), glycoprotein (G) and the RNA protease (also called the L-protein). (**B**) henipavirus genomes range from 18.2 kb (Nipah and Hendra viruses) to 19.9 kb (Melian virus) and encodes for It should be noted that multiple proteins are encoded in the phosphoprotein’s transcriptional unit, which also encodes for the C, W, and V-proteins. Thes3 proteins are expressed through different frameshifts in the transcriptional unit, which are caused by the insertion of either a single (V-protein) or double (W-protein) guanosine into the viral mRNA. The C-protein is expressed through an alternative open reading frame (ORF) of the transcriptional unit. Figure 1A,B were generated using modified icons and templates from Biorender.com (19 September 2023).

As with other members of the *Paramyxoviridae* family henipaviruses are enveloped, pleiomorphic viruses (Figure 1A) that are encoded by a single negative-stranded RNA encoding for 6 transcription units. As seen in Figure 1B, a prototypical henipavirus genome ranges from 18.2 kb (NiV and HeV) to 19.9 kb (Melian virus, MeliV), and encodes for 6 major structural proteins: nucleocapsid protein (N), phosphoprotein (P), matrix protein (M), fusion protein (F), glycoprotein (G) and RNA-dependent RNA polymerase (also called the L-protein) [4]. It should be noted that the phosphoprotein’s transcriptional unit also encodes for the C, W, and V-proteins, however, not all proteins are expressed by all henipaviruses [4]. The V and W proteins are expressed through different frameshifts in the transcriptional unit, which are caused by the insertion of either a single (V-protein) or double (W-protein) guanosine into the viral mRNA, whereas the C-protein is expressed through an alternative open reading frame (ORF) of the transcriptional unit. The C-protein may be involved in the budding of matrix proteins [5], as well as playing a role in shuttling between the cytoplasm and nucleus of the infected host cell [6]. V-, C-, and W-proteins have also been implicated in the regulation of the host interferon response [7,8].

### 1.3. Viral Tropism

Viral tropism can be analyzed at 3 different levels: (1) the host/species level; (2) the tissue/organ level; or (3) the cellular level. Each level requires broad study, due to the differing ability of each virus to demonstrate a unique ability to infect and/or cause virulence at each level. For example, while some viruses can be zoonotic, the extent to which they can cause disease may not be identical in all species; furthermore, their ability to induce pathology within the host is also not likely to be uniform, as certain tissues/organs are more prone to infection and pathogenesis. Finally, when histological analyses of infected organs are studied, often only certain cell types are susceptible, whereas others are not—this is most likely due to the differential expression of cellular receptors and/or due to events that occur downstream from the viral entry [9,10,11,12]. Therefore, this review will attempt to provide an overview of the most characterized henipaviruses, with tropism being discussed at all 3 levels.

### 1.4. Currently Well-Characterized Henipaviruses

#### 1.4.1. Hendra Virus (HeV)

HeV was first described in Hendra, a suburb of Brisbane (Queensland, Australia) in 1994, when a mare was found to be ill, ultimately dying 2 days later [3]. This was followed by the death of a companion horse, followed by other deaths within weeks of the first infected mare. During the first HeV outbreak, 14 horses and 1 human died in the Australian state of Queensland, with all cases associated with the same virus, as a morbillivirus cultured from the deceased human’s kidney was identical to another virus that had been isolated from the lung tissue from one of the deceased horses [2,3]. Vero E6 cells infected with the purified virus found that a cytopathic effect (CPE) could be observed, with multinucleated cells as a result of syncytial formation (an effect seen with other morbilliviruses) [1]. Furthermore, viruses amplified from Vero cell culture were then inoculated in recipient horses either intravenously or intranasally, with terminal disease being observed between 4- and 5-days post-infection (dpi) [1]. Purification of the virus also found its morphology to be consistent with that of other morbilliviruses. Finally, a partial sequence of the virus was obtained and found to be most similar to nucleotide sequences encoding for the matrix protein of bovine morbillivirus (MV-K1) and canine distemper virus (CDV) [1].

#### 1.4.2. Nipah Virus (NiV)

Between February and April 1999, severe cases of viral encephalitis were observed among pig farmers in Malaysia, affecting more than 200 people [13]. Of the affected individuals, 91 were admitted to hospital, where 28 deaths were recorded. The causative agent was suspected to be different from other viruses associated with encephalitis (i.e., Japanese encephalitis virus), as cases were clustered to members of the same household, thereby having a high degree of infectivity and pathogenicity. As the individuals were also known to have been in contact with pigs, it was suspected that this novel virus was transmissible from pigs to humans. Furthermore, following the mass culling of pigs in the outbreak areas, the incidence of the disease quickly decreased [14]. It was also shown that serum collected from infected individuals recognized Hendra virus, suggesting a degree of similarity between the two viruses. However, despite being serologically cross-reactive to HeV, the virus was originally found not to have significant pulmonary involvement, thereby giving its initial characterization of a Hendra-like virus [13]. Subsequent studies comparing the tropism of both HeV and NiV have demonstrated a high degree of similarity on a cellular level (as will be discussed later in this review).

#### 1.4.3. Cedar Virus (CedV)

Cedar virus was originally discovered in the Cedar Grove flying fox colony in Queensland, Australia during a study on the genetic diversity of HeV, with original isolates of CedV being isolated from urine samples of flying fox populations, which were then inoculated in primary cell lines derived from kidney, spleen, brain, placenta, as well as Vero cells [15]. CPE was observed in monolayers derived from kidney cells (PaKi cells), where syncytial formation by NiV and HeV infection had been previously observed. The virus was also able to cause CPE formation in Vero cells; however, the morphology of the CPE was different from that observed in HeV-infected Vero cells. Antibodies raised against CedV recognized HeV and NiV, but were non-neutralizing [15]. Genomic analysis of CedV demonstrated that it was most closely related to HeV and NiV, with genomic organization being highly similar to both viruses—with one notable exception; unlike HeV and NiV, CedV appeared to lack the RNA-editing mechanism required for the production of accessory proteins V and W expressed by the P-gene.

#### 1.4.4. Mojiang Virus (MojV)

As its namesake suggests, MojV was originally discovered in the Mojiang Hani Autonomous County in Yunnan Province (China), after 3 individuals developed an ultimately fatal form of pneumonia after working in an abandoned mine [16]. Half a year later, genomic studies were conducted in the mine, where swabs were taken from rats, bats, and shrews for virome analysis. From the tested swabs, the deduced nucleotide sequences were found to have the highest degree of identity with henipaviruses. Furthermore, these sequences were identified in 3 of the 9 rats analyzed [16].

#### 1.4.5. Ghana Virus (GhV)

Although an infectious, intact sample of Ghana virus has yet to be found in nature, the existence of this virus was determined through the elucidation of its genome during a screening of fruit bat (*Eidolon helvum*) droppings collected from a zoological garden in Kumasi, Ghana [17]. The initial characterizations made by Drexler et al. were based on the sequence of a 496 base pair amplicon, encoding for domains I and II of the polymerase gene (L-gene) [17], and were found to cluster to other known henipaviruses. Further genomic studies conducted by Drexler et al. [17] on bat and rodent samples from around the world identified 66 novel paramyxoviruses. Of these, partial sequences were obtained from at least 19 novel virus species associated with henipaviruses identified in African samples; and of these, a complete genomic sequence was obtained from one sample, identified as GH-M74a [17].

### 1.5. Other Viruses Not Currently Characterized by ICTV as of This Submission

#### 1.5.1. Langya Virus (LayV)

As of this submission, this virus has yet to be officially classified by ICTV, likely due to its very recent characterization in late 2022. The first description of this virus was made by Zhang et al., where sentinel surveillance of febrile illness of individuals with recent exposure to animals found 35 individuals with LayV infection in the Shandong and Henan provinces of China [18]. Through a combination of metagenomics analysis (from a throat swab sample of an infected individual), along with subsequent virus isolation, LayV was characterized as a henipavirus that is most closely related to Mojiang virus. Furthermore, a serosurvey of animals found that LayV was primarily found in shrews, suggesting that they might be the natural reservoir of this virus [18].

#### 1.5.2. Angavokely Virus (AngV)

Also discovered in 2022, AngV was identified from the urine of fruit bats in Madagascar [19], where a near-complete sequence (of more than 16 kb) was obtained. Although functional studies have yet to be performed, it was observed that the highly conserved residues associated with ephrin binding (i.e., NiV E533) are not present in AngV, thus suggesting the possibility of the use of another receptor for viral entry [19].

#### 1.5.3. Gamak Virus (GAKV)

Gamak virus was discovered through metagenomics analysis of shrews belonging to the *Crocidura* species [20] and was named based on its discovery in the Mount Gamak region of South Korea. Virus purification was then performed on the animals that had tested positive for GAKV and virus was amplified in Vero E6 cells, which was then used for subsequent in vitro infection assays. Phylogenetic analysis found that it was most similar to Mojiang virus. Furthermore, GAKV was able to infect human A549 cells and upregulate type I and III interferon-gamma genes, thus suggesting its ability to induce pro-inflammatory cytokines [20].

#### 1.5.4. Daeryong Virus (DARV)

Daeryong virus was also characterized in the same study conducted by Lee et al., along with GAKV. Originating from the Daeryong region of South Korea, DARV was also found to have a genome of more than 19.4 kb in length, making it slightly larger than other known henipaviruses [20]. As in the case of GAKV, phylogenetic analysis found DARV to be most similar to Mojiang virus.

#### 1.5.5. Melian Virus (MeliV)

Melian virus was recently discovered during a broad PCR-based screening study of paramyxoviruses in Belgium and Guinea described by Vanmechelen et al. [21], where virus RNA was extracted from the kidney of a shrew in Guinea. Phylogenetically, it was found to be most closely related to Daeryong virus, with a genome size of 19.9 kb [21].

#### 1.5.6. Denwin Virus (DewV)

**Figure 2:**
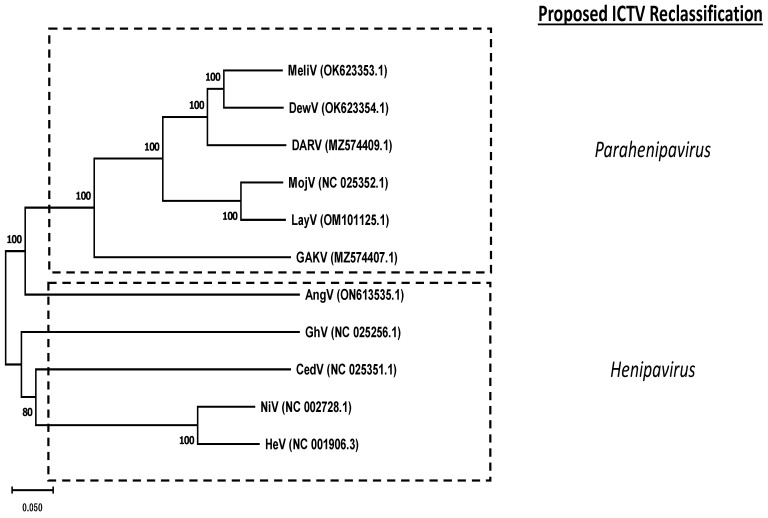
Phylogenetic relationships of the 11 characterized henipaviruses in this review. Amino acid sequences corresponding to putative L-proteins were aligned using ClustalW, with the resulting phylogenetic tree generated using the Neighbor-Joining method. The optimal tree is shown. The percentage of replicate trees in which the associated taxa clustered together in the bootstrap test (1000 replicates) are shown next to the branches. Genbank accession numbers are indicated in parentheses. Evolutionary analyses were conducted using MEGA11. The proposed reclassification of a novel “parahenipavirus” genus is indicated by hatched boxes.

Denwin virus was discovered in the same study by Vanmechelen et al., where its genome was extracted from the kidney of a killed shrew in Belgium [21]. As was the case with Melian virus, Denwin virus was also found to be most related to Daeryong virus, with a genome size of 19.7 kb [21]. A phylogenetic tree, based on the L-protein sequence of each virus is presented in Figure 2. While NiV and HeV are most closely related, CedV and GhV also cluster closely to the NiV/HeV branch. The more recently discovered henipaviruses (including those not yet classified by ICTV) display a greater degree of evolutionary divergence.

## 2. Experimental Host Tropism

### 2.1. Hendra Virus (HeV)

Apart from being the first member of the henipavirus to be identified, the first outbreak of the virus occurred in Australia in 1994, which affected more than a dozen horses, as well as a human [2,3,22,23].

#### 2.1.1. Hamsters

While humans and horses continue to be the main host targets for HeV, experimental infections have demonstrated that hamsters could also develop respiratory and neurological diseases [24]. Studies by Rockx et al. found that a high-dose intranasal infection (i.e., 10^5^ TCID_50_) could induce infection, with HeV infection being initiated primarily in the interstitium of the lungs. The respiratory epithelium was found to be an early target of infection, while endothelial cells were found to be infected in the later stages of the disease. Interestingly, this same study found that when a lower dose (i.e., 10^2^ TCID_50_) of HeV was administered intranasally, a slower, more systematic spread of the virus was observed, leading to the development of both respiratory and neurological disease. Furthermore, the presence of virus in both the blood and the central nervous system (CNS) suggested that this mode of infection induces disruption of the blood-brain barrier (BBB). Therefore, these results demonstrated that the clinical outcome of HeV infection in hamsters was dependent on the amount of virus used in the initial challenge dose.

#### 2.1.2. Non-Human Primates (NHPs)

The importance of demonstrating the pathogenicity of HeV in NHPs was described by Rockx et al. [25] as a model to measure the efficacy of antiviral treatments designed for humans. To this end, Rockx et al. infected African green monkeys (AGMs) intratracheally with 4 × 10^5^ TCID_50_ of HeV, then monitored for clinical signs over a course of 12 days. Of the 12 AGMs tested, 9 were treated with ribavirin either 24 h prior to challenge, 12 h post-challenge, or 48 h post-challenge. While the untreated AGMs required euthanasia between 7.5 and 9.5 dpi, AGMs treated with ribavirin either 24 h prior or 12 h post-infection required euthanasia within 10 and 10.5 dpi, a statistically significant delay of the onset of disease. Clinical signs included nasal discharge, labored breathing, and seizures. Pathology was also observed in the lungs, with pulmonary consolidation being seen in infected animals via radiology. Histological analysis also showed HeV tropism in the lung and brain; and viral replication was detected in the lungs, brain, liver, spleen, kidney, pancreas, lymph nodes, tonsils, and gastrointestinal organs [25]. A subsequent study by Bossart et al. using this same AGM model also found that a monoclonal antibody (m102.4), when administered within 72 h of HeV infection, could protect the animals from death [26].

#### 2.1.3. Guinea Pigs

The susceptibility of guinea pigs to HeV was demonstrated by Hooper et al., when HeV (then referred to as Equine Morbillivirus or EV) was inoculated subcutaneously using 5 × 10^3^ TCID_50_ [27]. 4 out of the 5 animals inoculated either died or became severely ill. Subsequent histological analysis demonstrated the virus in the lungs, heart, kidneys, liver, brain, and GI tract [27]. Further studies using guinea pigs were described by Williamson et al. [28] where pregnant guinea pigs (at 31–41 days gestation) were inoculated subcutaneously with HeV. Of the 18 animals inoculated, 7 animals died within 7 days of inoculation, with 8 more animals having to be euthanized before 15 dpi. Histological analysis determined that the virus was present in the lungs, kidney, brain, spleen, blood, placenta/uterus, and fetal tissue [29].

#### 2.1.4. Pigs

Although NiV has been extensively associated with pigs, little work has been performed to determine whether swine are also susceptible to the closely related HeV. Experimental infections in both 5-week-old Landrace pigs and 5-month-old Gottingen minipigs found that, after oronasal inoculation, both types of pigs developed respiratory signs at 5 dpi, with the minipigs developing neurological signs at 7 dpi [30]. Histological studies of the infected pigs confirmed the presence of the virus in the lymph nodes, tonsils, lung, and nasal turbinates. Furthermore, as HeV could also be detected in the olfactory bulbs, this finding suggested that HeV could invade the central nervous system via the olfactory nerves.

#### 2.1.5. Fruit Bats

While the initial outbreak of HeV was identified in humans and horses, serological studies in the areas surrounding the original outbreak in Queensland showed that fruit bats had antibodies that recognized the virus [31]. As other serological studies in the Queensland area found that fruit bats had antibodies against HeV, it was hypothesized that these animals could serve as a reservoir species. Therefore, studies were then conducted to determine if experimental subcutaneous or oronasal inoculation of fruit bats could induce clinical disease [32]. Of the 8 bats inoculated, 6 seroconverted, although none developed significant clinical signs (with only two bats showing vascular lesions), suggesting that bats are resistant to HeV-induced disease. Another subsequent study by Williamson et al. also confirmed that no overt clinical disease could be observed in HeV inoculated fruit bats [29,33].

#### 2.1.6. Cats

Shortly after the initial characterization of HeV in humans and horses, the susceptibility of cats to HeV was studied by Westbury et al. [34]. It was found that all cats inoculated either intranasally, subcutaneously, or orally developed HeV disease within 4–8 days, with death (or euthanasia being required) within 6–9 days. HeV was isolated from a variety of organs, including lung, trachea, brain, spleen, kidney, and lymph node [34]. This same study also demonstrated that infected cats could transmit the virus through contact to uninfected cats.

#### 2.1.7. Ferrets

As ferrets were previously found to be susceptible to NiV infection, HeV was also assayed in ferrets in a study by Pallister et al., where a recombinantly expressed HeV glycoprotein was assayed for its ability to confer protection [35]. In that study, ferrets were inoculated oronasally with different doses of HeV. In all animals, clinical signs were observed starting at 6 dpi, with all animals being euthanized by 9 dpi. Clinical signs included fever, depression, lack of grooming, and tremors. Histological analysis found the presence of HeV antigen in meningeal endothelial cells, bronchoalveolar endothelial cells, as well as cardiac, renal, splenic, pancreatic, and intestinal cells [35].

#### 2.1.8. Horses

As horses were among the first species to have been associated with HeV disease, attempts to experimentally replicate infection were performed by Mash et al. (2011). All 3 horses were infected oronasally with 2 × 10^6^ TCID_50_ of the virus [36]. 5–7 days later, the infected horses displayed clinical signs such as Fever, increased heart rate, depression, and reduced appetite [36]. 2 or the 3 horses exhibited nasal discharge starting from 2 dpi, with all horses requiring euthanasia between 6–9 dpi.

### 2.2. Nipah Virus (NiV)

Although discovered several years after the first known outbreak of HeV, NiV has become the most extensively studied member of the henipavirus family. As the original outbreak of NiV was associated with pigs and humans, experimental animal models quickly focused on trying to replicate the pathogenesis previously observed in natural outbreaks. Prior to discussing NiV tropism in animals, it should be noted that outbreaks in both Bangladesh and Malaysia differed with regards to pathogenicity and transmission, with Bangladeshi cases having a fatality rate of 75% [37], while the Malaysian cases had a fatality rate of 40% [38]. Furthermore, the two distinct strains, NiV-Bangladesh (NiV-B) and NiV-Malaysia (NiV-M), despite sharing nearly 92% nucleotide identity, displayed differing patterns of pathogenicity; for example, NiV-B was found to be associated with respiratory symptoms and a shorter incubation period [39] than its NiV-M counterpart [40]. The differences in pathogenicity were further analyzed in animal models such as non-human primates (NHPs), hamsters, ferrets and pigs. The different strains will be specified when discussing NiV experimental infection in the following section.

#### 2.2.1. Hamsters

As hamsters had already been found to be susceptible to HeV infection, their susceptibility to infection by the related NiV was assessed by Wong et al. [41]. In this study, Syrian golden hamsters were initially evaluated for the ability to develop disease following either intranasal or intraperitoneal inoculation with 10^7^ infectious viral particles. All hamsters died within 5–8 dpi [41]. In the next study, hamsters were inoculated with a lower dose (i.e., 10^4^ pfu) intranasally, and most animals succumbed to disease at 9–15 dpi, with clinical signs including imbalance, limb paralysis, limb twitching, and breathing difficulties. Reverse-transcription PCR (RT-PCR) performed following autopsy showed the presence of NiV in the brain, spinal cord, lung, kidney, spleen liver, and heart. Vascular pathology was also observed in these organs, with histological samples of the affected organs showing necrosis, inflammation, and multinucleation/syncytium formation. As these results are consistent with the pathology observed following human infection, it was proposed that hamsters were a suitable model for the study of NiV pathology.

In Syrian hamsters, NiV-M displayed a greater degree of virus replication, pathology and death compared to NiV-B-infected hamsters [42].

#### 2.2.2. Non-Human Primates (NHPs)

As in the case of HeV, the development of a non-human primate model for NiV was important, as NiV has been shown to be fatal in humans; and any validation of a vaccine or antiviral treatment against NiV in humans would ultimately have to be validated in NHPs. Such a model was described by Marianneau et al. [43] when squirrel monkeys (Saimiri sciureus) were inoculated either intravenously or intranasally with either 10^3^ or 10^7^ pfu. Clinical signs were observed between 7 and 19 dpi, which included: anorexia, weight loss, hyperthermia, acute respiratory syndrome, and uncoordinated motor movements, with some animals experiencing a loss of consciousness and coma [43]. Monkeys inoculated intravenously had more severe clinical signs, and required euthanasia earlier than those inoculated intranasally. While most intravenously inoculated monkeys displayed IgM- and IgG specific titers against NiV, most animals (of any experimental group) did not develop neutralizing antibody titers. Viral RNA was detectable in the liver, brain, kidney, lung, and lymph nodes. Histological examination also found the presence of virus in the lungs (alveolar walls), kidneys and brain. Another NHP model was described by Geisbert et al. [44], where AGMs were inoculated either intratracheally or intratracheally/orally, with NiV ranging from 2.5 × 10^3^ to 1.3 × 10^6^ pfu. All the AGMs tested displayed clinical signs, including depression, lethargy, open-mouthed breathing, loss of appetite, and loss of balance. All but one animal died (or required euthanization) by day 12 post-infection. Pathology associated with infection included: thrombocytopenia, severely inflated lungs, hemorrhages on the mucosal surface of the bladder, and excess blood-tinged pleural fluid. Presence of the virus was found in the spleen, lungs, and bladder [44]. Other studies using AGMs found that when the virus was administered intranasally using a laryngeal mask airway (LMA) mucosal atomization device (MAD), 2 × 10^3^ or 2 × 10^4^ pfu of NiV was sufficient to induce severe disease. All 4 monkeys tested required euthanasia by day 10 post-infection [45]. Finally, an interspecies comparison between AGMs and macaque monkeys found that macaques were more likely to survive NiV infection than AGMs [46]. All macaques that were inoculated both intratracheally and intranasally with 5 × 10^5^ pfu of NiV survived infection until the end of the study (which contrasted with AGMs, which usually succumb to infection within 5–7 dpi). Furthermore, all macaques seroconverted and generated neutralizing antibody titers comparable to AGMs. This same study also found a correlation between the surviving animals and T-helper cell profiles that skewed towards the Th1 subtype [46]. Other non-human primates, such as marmosets, were also found to be susceptible to NiV infection. Studies by Stevens et al. [47] demonstrated that these animals can also serve as an animal model for viral studies. When 6.3 × 10^4^ pfu of NiV was administered intratracheally and intranasally, all 4 inoculated marmosets developed clinical signs, and required euthanasia between 8 and 11 dpi [47]. Pulmonary edema and viral hepatitis were observed post-mortem, with syncytial formation being observed in the pulmonary tissue. Histopathological analysis also found the presence of viral antigen in pulmonary and cardiac tissue [47].

With regards to differences in pathogenicity between NiV-B and NiV-M, it was found that in African green monkeys (AGMs), NiV-B was uniformly lethal, while only 50% of AGMs infected with NiV-M succumbed to infection [48].

#### 2.2.3. Guinea Pigs

Although Wong et al. did not observe NiV pathology in guinea pigs [41], other studies did observe some clinical signs following infection. Middleton et al. inoculated 8 guinea pigs with 5 × 10^4^ TCID_50_ intraperitoneally, with 3 out of 8 animals displaying abnormal behavior and ataxia between 7 and 8 dpi [49]. Following euthanasia, NiV was isolated from the heart, lung, uterus, spleen and blood of the infected animals. Another study by Torres-Velez et al. found an even greater incidence of NiV pathology in guinea pigs, with nearly all animals succumbing to infection between 4 and 8 dpi after intraperitoneal inoculation with 6 × 10^4^ pfu of virus [50]. Viral antigen was detected over a wide variety of tissues and cell types, with syncytial formation also being observed.

#### 2.2.4. Pigs

As the original outbreak of NiV was linked to pig farms and pig trade, a more extensive study of the pathology and transmission of the virus to pigs was required. Experimental infection of NiV in 6-week-old piglets was performed by Middleton et al. [51], where 3 pigs were inoculated orally, while another 3 were inoculated subcutaneously with 5 × 10^4^ TCID_50_ of NiV (virus that had not been passaged in cell culture). The remaining 2 piglets were not inoculated with the virus, but remained in direct contact with the other pigs. Over the course of 21 days, all animals were analyzed for pathology, clinical signs, as well as the presence of virus in different samples taken. Of the 4 pigs inoculated subcutaneously, 2 required euthanasia between 7 and 8 dpi, due to severe clinical disease with ataxia, semi-consciousness and lack of coordinated movements. However, while another pig also showed some clinical signs, it was able to recover. Interestingly, all of the orally infected pigs showed little to no clinical signs and survived the entire 21-day study [51]. Organs recovered from the animals showed that NiV could be recovered from tonsil, nose, blood, lung and spleen. Pathological analysis showed vasculitis and degeneration of pulmonary blood vessels, with syncytial cell formation observed within lymphoendothelial cells. Although the contact animals did not show significant clinical signs, the virus was recovered from their tonsil and nose. Seroconversion was observed in both groups, with neutralizing antibodies detected between 14 and 21 dpi. Another study by Weingartl et al. [52] using 5-week-old piglets found similar results when using the oronasal inoculation route, as most pigs were clinically healthy throughout the study, with only 2 (out of 11 pigs) requiring euthanasia due to severe clinical signs. As in the previous study, the presence of the virus was detected in both healthy and diseased pigs; however, their findings also demonstrated that NiV replication occurred in the CNS prior to infection of the endothelial cells of the blood/lymphatic system. Their results represented the first demonstration that NiV could cross the BBB of pigs. The conditions used in this experimental setup were then used for subsequent studies of possible immunosuppression of the infected pigs leading to spread of bacteria [53], as well as the evaluation of multiple vaccine candidates [54,55]. In pigs experimentally infected with with NiB-B, no clinical signs were observed and no viremia was detectable at any sampling date. However, isolation of virus was successful from several tissues (olfactory bulb, turbinates, trachea, lung, submandibular LN, tonsils, and meninges) and nasal washes indicating virus replication [56].

#### 2.2.5. Cats

Following the initial outbreak of NiV in Malaysia in the late 1990s, it was suspected that the virus could infect companion animals such as dogs and cats [57]. The experimental infection of cats was demonstrated by Middleton et al. [51], where 2 cats were inoculated oronasally with 5 × 10^4^ TCID_50_ of virus. Clinical signs and rectal temperatures were assessed daily for 21 days. While both cats appeared normal for the first 4 days, both cats developed fever by day 6, with the animals showing signs of depression and increased respiratory rates [51]. Febrile illness, depression and breathing difficulties continued until one of the two cats required euthanasia at 9 dpi, while the other cat recovered. Virus was isolated from the blood, tonsil and urine. Another study performed by Mungall et al. [58] showed that clinical signs could be observed when NiV was administered subcutaneously at either dose of 5 × 10^2^ or 5 × 10^3^ TCID_50_. Both experimental groups demonstrated febrile illness until being euthanized at 9 dpi. Upon necroscopy, the cats were found to have hemorrhagic nodular lesions on the visceral pleura. Histological analysis found NiV-positive hemorrhagic and necrotic lesions of pulmonary tissue, including syncytial formation [59]. These experimental conditions served as the basis for the validation of a NiV subunit vaccine, encoding for the soluble G protein of HeV [58,59]. In one study, following subcutaneous administration of 5 × 10^2^ TCID_50_ of virus, a cat was found to be pregnant during necropsy [60]. Postmortem samples of placenta and fetal tissues tested positive for the virus, and virus could be re-isolated from the placenta [60], thereby demonstrating the ability of NiV to be vertically transmitted.

#### 2.2.6. Bats

As bats were suspected of acting as a reservoir species from the earliest outbreak for NiV [61], the susceptibility of native Australian flying foxes (*Pteropus poliocephalus*) was evaluated by Middleton et al. [49], where 17 grey-headed flying foxes were inoculated subcutaneously with 5 × 10^4^ TCID_50_ of NiV and monitored for 21 days, with body temperature and bodyweight being measured daily. Serum samples were also collected at various time points. The infected bats presented subclinical infection characteristics, as evidenced by the presence of viral antigen in selected viscera and seroconversion. Although the virus was not recovered from the wide variety of organs seen in other animal models, NiV was recovered from the kidney of one male bat, as well as the uterus of one female bat [49]. Furthermore, NiV was isolated from the urine of one animal. Pathology studies did not display any gross abnormalities. All tissue samples tested negative for NiV through immunohistochemical labelling.

#### 2.2.7. Ferrets

As in the case of HeV, ferrets were also found to be susceptible to NiV. A ferret animal model for NiV infection was described by Bossart et al. [26], where a human monoclonal antibody m102.4 was assessed for its ability to protect against lethal disease caused by NiV. In this model, ferrets were inoculated oronasally with titers varying from 5 × 10^1^ TCID_50_ to 5 × 10^4^ TCID_50_. While clinical signs were observed in at least one animal inoculated with 5 × 10^2^ TCID_50_, viral shedding and disease were consistently observed at 5 × 10^3^ and 5 × 10^4^ TCID_50_. Clinical signs were found to be consistent to those found in humans, including loss of appetite, depression, dyspnea and neurological disease and generalized vasculitis (including syncytia formation of the epithelium) [26]. The presence of NiV antigen was detected in a variety of organs, including the pharynx, blood, kidney, liver, rectum, spleen and brain. The ferret model was used to validate vaccine candidates such as recombinant HeV G [62], as well as recombinant vesicular stomatitis virus (VSV) expressing either the F- or G-proteins of NiV [63].

In ferrets, a higher degree of viral shedding and tissue tropism was found in NiV-B, compared to NiV-M; and higher levels of RNA were found in the NiV-B strain than its Malaysian counterpart [64].

### 2.3. Cedar Virus

Cedar virus (CedV) was originally isolated from fruit bats in Australia [11], with attempts to evaluate its ability to infect other animal models associated with henipavirus infection being evaluated by Marsh et al. [15].

#### 2.3.1. Guinea Pigs

Using CedV that was originally isolated from the urine of flying foxes, followed by two serial passages in bat PaKi cells, 2 × 10^6^ TCID_50_ of CedV was inoculated intraperitoneally in guinea pigs [15]. Although no clinical signs were observed over 21 days, virus-specific antibodies were generated, as neutralizing antibodies were detected in 2 out of the 4 guinea pigs tested.

#### 2.3.2. Hamsters

CedV was also found to experimentally infect hamsters. Schountz et al. [65] demonstrated that virus replication could be observed following intranasal inoculation of the virus (10^5^ TCID_50_), with replication occurring in the lungs and spleen of the animal.

#### 2.3.3. Mice

In the same study by Marsh et al. [15], BALB/c mice were also inoculated oronasally with 1 × 10^5^ TCID_50_ of CedV and monitored for 21 days. None of the mice displayed any clinical signs, nor were any neutralizing antibodies detected.

#### 2.3.4. Ferrets

In the study previously discussed [15], ferrets were also inoculated oronasally with 2 × 10^6^ TCID_50_ of CedV and monitored for 21 days. Although no clinical signs were observed, neutralizing antibodies were detected by 10 dpi. However, hyperplasia of tonsillar and bronchial lymphoid tissues was observed, with virus being detected in the bronchial lymph nodes [15].

## 3. Tissue/Organ Tropism

### 3.1. Hendra Virus (HeV)

Following the outbreak of HeV in Queensland in 1995, further investigation was conducted on the infected horses, and subsequent post-mortem analysis showed edema and congestion in the lungs, with syncytial formation in the vascular endothelium [1,2,3]. An autopsy performed on the deceased human found that both lungs were congested, hemorrhagic and filled with fluid. Histology performed on the lungs demonstrated necrotizing alveolitis, with giant cells, viral inclusion and syncytial cells [1,2,3].

In-depth experimental infections in horses following oronasally HeV administration lead to systemic infection of the animal and inducing pathology consistent with that reported in the initial HeV outbreak [36].

As fruit bats were found to be a natural reservoir for transmission of HeV to horses, multiple studies looked into the tissue tropism of HeV in the Australian fruit bat genus *Pteropus*. RT-qPCR performed on tissue samples from 300 archived bats in the Queensland area during 1996–1997 detected HeV in more than 6% of the animals, with the virus being detected predominantly in the spleen, but also from lung, kidney, liver and placenta [66]. It should also be noted that HeV has also been detected in bat secretions such as urine [67].

### 3.2. Nipah Virus (NiV)

The tropism associated with NiV was immediately characterized in 3 pig farmers in Malaysia who exhibited symptoms consistent with viral encephalitis [13,68]. All 3 individuals exhibited fever and headache, rapidly deteriorating into hypotension and death. Cerebrospinal fluid was collected from all 3 patients prior to their death and was submitted to virus isolation. ELISAs were also performed, which ultimately confirmed the presence of NiV-specific antibodies. When histopathological analysis was performed on all 3 individuals, endothelial damage and vasculitis (mainly in the arterioles, capillaries and venules, as well as the muscular arteries) was observed. Blood vessels were characterized by vessel wall necrosis, thrombosis, and inflammatory-cell infiltration of neutrophils and mononuclear cells [13].

The brain was found to be the most severely affected organ, with eosinophilic and nuclear viral inclusions being observed [13], which was found to be consistent with other paramyxovirus infections. Syncytial cell formation was also seen in the brain, lungs and Bowman’s capsule of the glomerulus. The main cause of death was most likely due to widespread focal infarction of the brain, with the possibility of direct infection of the neurons [13].

In vitro infections using a variety of cell lines confirmed broad NiV tropism. Brain-derived endothelial cells (either primary or immortalized) were found to be permissive to NiV infection [68]; specifically, human brain microvascular endothelial cells (HBMECs) and primary porcine microvascular endothelial cells (PBMECs) that were freshly isolated from pigs were found to be susceptible to NiV infection and replication in vitro. This same study also demonstrated that non-permissive cells such as porcine aortic endothelial cells (PAECs), when transiently expressing ephrinB2, can be rendered permissive to NiV infection. As ephrinB2 is readily expressed in vascular tissue, the combined results confirm the susceptibility of vascular endothelial cells to NiV infection [69].

Studies using Syrian hamsters oronasally infected with either a Bangladeshi or Malaysian isolate of NiV found that both isolates caused rhinitis and bronchointerstital pneumonia within 2 dpi [70]. Immunohistochemistry found that NiV exhibited endothelial tropism in the small and medium caliber arteries and arterioles, but not the veins in the lung. The authors hypothesized that this distribution correlated with the expression of ephrinB2 in the arterial, but not in the venous, epithelium [70].

A more in-depth study of intranasally inoculated Syrian hamsters found that NiV replication could be detected in the lung and nasal turbinates (where the respiratory and olfactory epithelium are located) within 8 h of experimental inoculation [71]. Immunohistochemistry performed on tissues from the lung found that NiV could be detected in type I pneumocytes, bronchiolar respiratory epithelium and alveolar macrophages within 8 h; and by 16 h post-infection, NiV was shown to disseminate from the bronchiolar respiratory epithelium to the underlying bronchiolar smooth muscle, with further dissemination into the arterial smooth muscle cells being observed between 32 to 48 h post-infection [71]. Based on these results, the authors hypothesize that the initial infection of the nasal turbinates precedes NiV spread to the brain while dissemination into the arterial smooth muscle cells leads to viral dissemination into the vasculature. It should be noted that another study confirmed that NiV can replicate in human smooth muscle cells; however, no cytopathogenicity was observed in these infected cells [72].

Another method in evaluating NiV tropism in Syrian hamsters was described by Welch et al. [73], where recombinant NiV expressing the fluorescent protein ZsGreen1 (cloned into the M gene of the NiV genome) was found to be localized to the lung, brain, liver, nasal turbinates and kidney of infected animals.

NiV infectivity in the vascular system was further corroborated by Ang et al. [74], where human pluripotent stem cells were differentiated into either artery or vein endothelial cells in vitro. Upon generating two distinct, highly enriched cell populations, the cultures were then infected with either NiV or HeV. In both cases, syncytial formation was observed to be more than 11 times greater in the artery-differentiated cells compared to the vein-differentiated cells. Furthermore, both cell populations, when deleting the ephrinB2 gene using Cas9, showed no susceptibility to NiV and HeV infection, thereby demonstrating the importance of ephrinB2 in the tropism of both viruses [74].

When studying peripheral blood mononuclear cells (PBMCs) of pigs, Stachowiak and Weingartl [75] demonstrated that NiV replication could be detected in monocytes, CD6^+^CD8^+^ T lymphocytes and NK cells. The authors proposed that, as CD6 is a ligand for CD166 (an adhesion molecule expressed on microvascular endothelial cells of the blood-air and blood-brain barrier), as this could explain the link between infection and vasculitis in small blood vessels.

## 4. Cellular Tropism

Paramyxoviruses have been previously shown to require both the cell-attachment glycoprotein (designated as either the hemagglutinin/H-protein, hemagglutinin-neuraminidase/HN-protein or the glycoprotein/G-protein, depending on the family of paramyxovirus being studied), as well as the fusion protein (F-protein) for entry into the host cell [76,77].

### 4.1. Nipah Virus (NiV) and Hendra Virus (HeV)

The first confirmation that a G-F protein complex was required for henipavirus entry was demonstrated by Bossart et al. [78], where cell lines were transfected with plasmids encoding for HeV F-protein, G-protein, or a combination of both. It was observed that syncytial formation (i.e., the multinucleated cells) only occurred when both plasmids were transfected. Furthermore, recombinant vaccinia virus expressing these 3 constructs also showed that syncytial formation in HeLa cells required both F- and G-proteins [78]. This study also demonstrated that syncytium formation could be observed using cells from bats, horses, cats, pigs, rabbits, monkeys, mice and ducks indicating a broad host spectrum.

Comparable studies with NiV F and G protein showed similar results [79], demonstrating that both F- and G-protein are required for the induction of cell-fusion. Furthermore, it was found that both NiV glycoproteins demonstrated heterotypic functional activity with their respective HeV counterparts. This degree of heterotypic activity was only observed between NiV and HeV, as envelope glycoproteins from measles virus (MV) and canine distemper virus (CDV) could not be interchanged with NiV or HeV. These findings suggest a high degree of functional conservation between NiV and HeV envelope proteins which is not retained across other genera (i.e., morbillivirus) in the paramyxovirus family.

### 4.2. EphrinB2 and –B3 Are Host Receptors for NiV and HeV

Upon the observation that syncytium formation was detected after transient expression of F- and G-proteins, experiments to determine the precise host-receptors that interact with HeV and NiV envelope proteins were performed by Bonaparte et al. [80]. Having previously observed that a HeLa cell-line derivate (HeLa-USU) was non-permissive to syncytium formation following NiV and HeV expression, this cell-line was compared to NiV and HeV permissive cell-lines by microarray analysis; these included HeLa-CCL2, the human glioblastoma cell line U373, and the human head and neck carcinoma PCI-13. Potential gene candidates were identified based on known or predictive plasma membrane surface-expressed proteins. Of the 10 that were identified, ephrinB2 (EFNB2) was found to permit NiV and HeV infection, based on the ability of EFNB2 to bind to soluble HeV and NiV glycoprotein in ELISA assays, as well as permitting NiV and HeV infection when expressed in HeLa-USU cells [80].

Experiments using the closely related ephrin-B1 receptor demonstrated that this receptor did not mediate entry into entry of NiV F/G pseudotype viruses [81], indicating receptor specificity. However, following studies using recombinant proteins encoding for all known ephrins (i.e., ephrinA1–A5 and ephrinB1–B3) found that ephrinB2 and ephrinB3 interacted with NiV-G, as detected by surface plasmon resonance [82]. Furthermore, experiments using pseudotype VSV (expressing NiV envelope proteins) showed that soluble ephrinB2 and ephrinB3 competitively inhibited interactions with CHO cells expressing either ephrinB2 or ephrinB3, suggesting that NiV-G interacts with both receptors through an overlapping site [82]. Mutational analysis further located this site to two residues, Leu124-Trp125, located in the G-H loop of both ephrinB2 and ephrinB3 that are critical for NiV binding and entry [82].

Guillaume et al. provided further insight into the critical residues of NiV G which were required for binding to ephrinB2 [83]. In that study, plasmids encoding for NiV G were constructed with point mutations of fifteen charged residues within the globular head of NiV G, and then analyzed in cell fusion assays. Mutation of 7 residues, W504, E505, N557, Q530, T531, A532, and E533, reduced fusion, with the E533 mutation being responsible for the highest decrease in fusion capacity. The critical relevance of E533 for receptor binding was further supported by the finding that E533 was replaced in an escape mutant.

Studies using soluble NiV and HeV G proteins found that while both proteins interacted with ephrinB2 with similar affinities, soluble NiV G protein bound to ephrinB3 with an approximately 30-fold higher affinity than HeV G [83]. As the globular domain of both proteins differ only at amino acid residue 507 (i.e., HeV Ser507, NiV Thr507), a recombinant soluble HeV G protein was constructed, substituting Ser507 for Thr507 (i.e., S507T). This mutation conferred HeV G-binding affinity to ephrinB3 to a level comparable to NiV G [84]. As these results suggested that Thr507 plays an important role in the affinity of NiV G for ephrinB3, additional experiments were performed using NiV G with amino acid substitutions previously implicated by Guillaume et al. [82] for ephrinB2. While most of the mutants (with the notable exception of E533Q) did not show decreased affinity for ephrinB2, all of the assayed mutants did show decreased affinity for ephrinB3, suggesting that the interaction of NiV G with both receptors is distinct [84].

As ephrinB2 is expressed on endothelial cells, neurons, and smooth muscle cells [85,86], while ephrinB3 is expressed in the central nervous system [87], the tissue distribution of these molecules overlaps strongly with the organ tropism associated with NiV infection.

### 4.3. Specificity of Other Henipavirus Envelope Proteins in Cellular Tropism

#### 4.3.1. GhV

While NiV and HeV are the most extensively studied, the characterization of other henipavirus genomes have led to the study of cell tropism, using a strategy of expressing the putatively encoded envelope proteins in cells. Using bat and human serum that displayed neutralizing activity for NiV, Pernet et al. [88] demonstrated that this serum could neutralize pseudo particles expressing GhV envelope glycoproteins. An example of this was described by Krüger et al. [89], where the envelope proteins of GhV were cloned into an expression plasmid and assayed for syncytial formation. When transiently expressed in BHK 21, Vero76 and HypNi/1.1 cells (a kidney cell line derived from the hammer-headed fruit bat Hypsignathus monstrosus), syncytium formation was only observed in HypNi/1.1 cells; although it should be noted that the number of nuclei per syncytium was considerably less than that observed for HeV and NiV. This suggested that GhV has a narrower range of cell tropism compared to HeV and NiV. However, this same study also demonstrated GhV G protein’s interaction with ephrinB2, and that syncytium formation was pH dependent most likely due to the requirement of an acidic pH in the endosomal compartment for henipavirus fusion protein proteolytic cleavage [90,91,92]. These findings suggested that despite its more limited range of cell tropism, GhV envelope proteins may interact with the host cell in a manner similar to its NiV and HeV counterparts. These findings were further expanded upon, with another study on the host-restrictive properties of GhV envelope proteins [93]. In this study, two other cell lines derived from bats (i.e., chiropteran) were transfected with plasmids encoding for GhV F and G proteins, with all chiropteran cell lines showing increased surface expression of G-proteins (compared to non-chiropteran cell lines). Interestingly, when the G proteins of both GhV and NiV were expressed in the same chiropteran cell lines, NiV G protein exhibited a significantly greater level of surface expression compared GhV G protein, suggesting that these differences in surface expression may explain the more limited syncytium formation observed by GhV envelope proteins [93].

The host cell specificity associated with GhV envelope proteins was also expanded upon by a study from Weis et al. [94]. Here, heterotypic expression of the envelope protein (i.e., the co-expression of NiV G with GhV F-protein) in Vero cells did not result in syncytium formation; however, when GhV G-protein was co-expressed with NiV F-protein, syncytium formation was observed (albeit with a reduced number of nuclei/syncytium), thereby suggesting that the limited host range of GhV may be due to the F protein. Furthermore, when pulse-chase experiments were performed on both GhV and NiV F proteins expressed in MDCK cells, it was found that relatively little GhV F protein was cleaved into its biologically active form (i.e., F_1_ and F_2_) compared to NiV, suggesting a possible mechanism behind GhV F protein’s limited host range [94].

These results were also confirmed by Lawrence et al. [95] when comparing GhV and NiV envelope proteins in another chiropteran cell line EidNi (a kidney cell line derived from the straw-colored fruit bat *Eidolon helvum*). As was the case in the study of Weis et al., GhV envelope proteins induced syncytium formation in EidNi, but not in 293T, HeLa or Vero cells. Heterotypic assays using combinations of NiV and GhV G- and F-proteins also yielded identical results to those presented by Weis et al.

#### 4.3.2. Mojiang Virus (MojV)

Rissanen et al. demonstrated that MojV G protein is antigenically distinct from NiV and HeV; however, recombinant expression of MojV F and G protein did induce syncytium formation in human cells lines A549, U87 cells, along BHK and HEK293T cells [96]. This same study also demonstrated (through heterotypic expression of MojV F and G proteins with their NiV counterparts) that MojV envelope proteins do not interact with human ephrinB2 or B3.

#### 4.3.3. Cedar Virus (CedV)

A study by Pryce et al. using HEK293T cells found that CedV G protein displayed high affinity for human ephrin B2, while showing little affinity for ephrin B3. More interestingly, CedV G protein also displayed an affinity for human ephrin B1 [97]. These results were further confirmed when CHO cells expressing recombinant ephrin B1 or B2 enabled entry of CedV pseudotyped virus, while CHO cells expressing ephrin B3 did not [97]. Furthermore, Laing et al. demonstrated that CedV glycoproteins displayed a unique affinity for ephrin receptors [98]. Using cell-cell fusion assays between CHO745 cells expressing CedV F/G proteins and ephrin-expressing target cells, they found that fusion could be observed with target cells expressing ephrinA1, A2, A5, B1 and B2 (with the latter two showing the greatest degree of fusion). It should also be noted that CedV glycoproteins had species specific affinity for mouse ephrin A1 [98].

**Figure 3:**
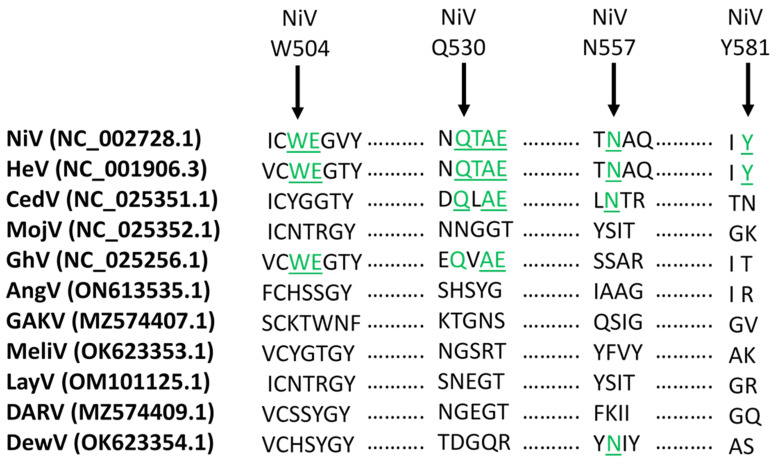
Alignment of henipavirus ephrin binding residues. Residues of the G-protein previously associated with ephrin binding are indicated in green, and include W504, E505, Q530, T531, A532, E533, and Y581 (NiV numbering). Genbank accession numbers are indicated in parentheses. This figure is modified from Madera et al. (Madera et al., 2022), including the sequences of additional henipaviruses.

As tropism appears to be strongly linked to ephrin receptors, a summary of the putatively conserved ephrin binding domains associated with the G-proteins of the previously discussed members of this genus is shown in Figure 3. The lack of conserved residues in MojV, AngV, GAKV, MeliV, LayV, DARV, and DewV strongly suggests that these viruses will likely display different binding affinities to ephrin B2 and B3.

A more in-depth description of ephrin receptor-G protein interactions is described in reviews by Pernet et al. [12] and Bhattacharya et al. [99].

## 5. Conclusions

While the initial characterization of henipaviruses was described less than 30 years ago, with the identification of Hendra and Nipah viruses. The discovery of these two viruses occurred as a result of lethal pathologies observed associated with their emergence; in the case of Hendra, the initial outbreak was associated with lethal infections in horses and a human; while in the case of Nipah, lethality was associated with domesticated pigs and humans. If one were to have made a general characterization of this genus following the discovery of HeV and NiV, one could have easily proposed that henipaviruses are a strongly pathogenic genus, and predicted that the discovery of future species would also likely pose a threat to human and veterinary health; and while another lethal member of this genus was identified following HeV and NiV (i.e., MojV), other members of this genus, including CedV and GhV, have yet to be associated with severe illness or death.

Furthermore, the use of large-scale genomics has elucidated several “henipa-like” genomes, which ICTV has proposed to be categorized as “parahenipaviruses”. According to their current proposal, “Specifically, members of the rodent-/shrew-borne clade, now recognized as the genus “*Parahenipavirus*”, have an extra ORF contained within the F gene that encodes a transmembrane protein.” (https://ictv.global/filebrowser/download/12884 accessed on 17 August 2023). Phylogenetically, there is some degree of agreement with the proposed taxonomic changes, as the proposed “parahenipaviruses” cluster from the previously characterized henipaviruses (i.e., NiV, HeV, CedV, GhV and AngV) (Figure 2). As meta-genomic analysis continues to become more accessible, one should expect that the members of this genus will continue to grow. A summary of our current understanding of henipavirus tropism is summarized in Table 1, while the experimental conditions used to study animal models of henipavirus are summarized in Table 2.

## Figures and Tables

**Table 1:** A summary of henipavirus tropism.

Virus	Organisms	Organ/Tissues	Cells
Hendra (HeV)	-Natural hosts: horses, humans, bats -Experimental: hamsters, guinea pigs, cats, ferrets, pigs, non-human primates	-lung, kidney, liver, placenta, lymph nodes, vascular endothelium	-primary epithelial cells -cell lines: HeLa, 293T, 3T3, BSC-1, HuTK^−^143B, Vero
Nipah (NiV)	-Natural hosts: pigs, humans, bats -Experimental: hamsters, guinea pigs, cats, pigs, non-human primates, ferrets	-lung, brain, liver, arteries, bronchiolar respiratory epithelium, nasal turbinates, kidneys	-primary: epithelial cells (artery), macrophages, endothelial cells, neurons -cell lines: HeLa, 293T, CHO, U87, U373, PCI-13, Vero
Cedar (CedV)	-Natural hosts: bats -Experimental: guinea pig, mice, ferrets, hamster	-lungs, spleen, also isolated from urine	-cell lines: PaKi, A549, HEK293T, Vero, BHK21, L2, C6, Rat2 cells
Ghana (Kumasi/M74 virus)	-Natural hosts: bats	NA	-cell lines: HypNi/1.1, EidNi
Mojiang (MojV)	-Natural hosts: humans, rats	NA	-cell lines: A549, U87, BHK21, HEK293T, Vero, Hep2
Langya (LayV)	-Natural hosts: humans, shrews, voles	-NA	-cell lines: Vero
Angavokely (AngV)	-Natural hosts: bats	-unclear (isolated from urine)	NA
Gamak (GAKV)	-Natural hosts: shrews	-kidney	-cell line: Vero E6
Daeryong (DARV)	-Natural hosts: shrews	-kidney	NA
Denwin (DewV)	-Natural hosts: shrews	-kidney	NA
Melian (MeliV)	-Natural hosts: shrews	-kidney	NA

**Table 2:** Animal models of henipavirus infection.

Virus	Host Species	Viral Dose Used	Inoculation Route	Symptoms/Outcome of Infection	End of Study (P)/Death or Euthanasia (E)	Reference
Hendra virus	Hamster	10^5^ TCID_50_ 10^2^ TCID_50_	i.n. i.n.	Acute respiratory distress Neurological signs, systemic spread	3 days (E) 6 and 7 days (E)	[41,42]
	African green monkeys (AGM)	4 × 10^5^ TCID_50_	i.t.	Nasal discharge, labored breathing, seizures	7.5–9.5 days (E)	[25,26]
	Guinea pigs	5 × 10^3^ TCID_50_	s.c.	Clinical ill	8 to 13 days (E); 1 until P	[27]
	Pigs	6.6 × 10^7^ PFU	o.n.	Respiratory distress	5 days (E)	[30]
	Fruit bats	5 × 10^4^ TCID_50_ 5 × 10^4^ TCID_50_	s.c. o.n.	No significant clinical signs	P	[29,32]
	Cats	10^3.6^ TCID_50_ 10^3.6^ TCID_50_ 10^3.6^ TCID_50_	i.n. s.c. o.	Fever, depression, increased/variable respiratory rate	6–9 days (E)	[34]
	Ferrets	5 × 10^3^ TCID_50_	o.n.	Fever, depression, tremors	9 days (E)	[35]
	Horses	2 × 10^6^ TCID_50_	o.n.	Fever, increased heart rate, depression, reduced appetite, nasal discharge	6–9 days (E)	[36]
Nipah virus (Malaysia; NiV-M)	Hamster	10^2^ TCID_50_ 10^7^ TCID_50_ 10^4^ PFU	i.n.i.n. i.p. i.n.	Respiratory signs Imbalance, breathing difficulties, limp paralysis	5 days (E) 9–12 days (E) 5–8 days (E) 9–14 days (E)	[24] [41]
	Non-human primates Squirrel monkeys African green monkeys Macaque monkeys	10^3^/10^7^ PFU 10^3^/10^7^ PFU 2.5 × 10^3^ to 1.3 × 10^6^ PFU 2 × 10^3^ or 2 × 10^4^ PFU 5 × 10^5^ PFU 5 × 10^5^ PFU	i.v. i.n. i.t. i.t./o. i.n. i.t. i.n.	Anorexia, acute respiratory distress, uncoordinated motor movements up to coma Depression, lethargy, breathing difficulties, loss of appetite, imbalance No clinical disease	8, 12, 21 days (E) P 10–12 days (E) 9–10 days (E) P	[43] [43] [44] [45] [46]
	Guinea pigs	5 × 10^4^ TCID_50_ 6 × 10^4^ PFU	i.p. i.p.	Abnormal behaviour, ataxia (3/8 animals)	7/8 days (E) 4 to 8 days (E)	[49] [50]
	Pigs	5 × 10^4^ TCID_50_ 5 × 10^4^ TCID_50_ 2.5 × 10^5^ PFU	o. s.c. o.n.	No clinical signs Ataxia, semi-consciousness, uncoordinated movement 2/11 animals severe disease	P 7/8 days (2 out of 4 animals) (E) 2 out of 11 (E)	[51] [52]
	Fruit bats	5 × 10^4^ TCID_50_	s.c.	Subclinical	P	[49]
	Cats	5 × 10^4^ TCID_50_ 5 × 10^2^ TCID_50_ 5 × 10^3^ TCID_50_	o.n. s.c.	Fever, increased respiratory rateFebrile illness	9 days (E), 1 of 2 animals 9 days (E)	[51] [58]
	Ferrets	5 × 10^1^ TCID_50_ to 5 × 10^4^ TCID_50_	o.n.	Loss of appetite, depression, dyspnea, neurological disease	6–10 days (E), depending on dose	[26]
Nipah virus (Bangladesh, NiV-B)	Pigs	2 × 10^5^ PFU	o.n.	No clinical signs	P	[56]
	NHPs African green monkeysMarmorsets	2 × 10^3^ or 2 × 10^4^ PFU 6.3 × 10^4^ PFU	i.n. i.t. i.t. and i.n.	Fever, depression, loss of appetite, labored breathing Weight loss, multiple organ dysfunction	9–10 days (E) 8–11 days (E)	[48] [47]
	Hamster	1 to 10^5^ TCID_50_ 10^5^ TCID_50_	i.p. i.n.	Respiratory stress with higher doses; neurological signs with lower doses	8–9 days (NiV-M) (E) 8–14 days (NiV-B) (E)	[42]
	Ferret	5 × 10^3^ TCID_50_	o.n.	Respiratory and neurological signs	7–9 days (E)	[64]
Cedar virus	Mice (BALB/c)	1 × 10^5^ TCID_50_	o.n.	No clinical signs	P	[15]
	Guinea pigs	2 × 10^6^ TCID_50_	i.p.	No clinical signs	P	[15]
	Hamsters	1 × 10^5^ TCID_50_	i.n.	No clinical signs	P	[65]
	Ferrets	2 × 10^6^ TCID_50_	o.n.	No clinical signs	P	[15]

i.n., intranasal; i.t., intratracheal; s.c., subcutaneous; o.n., oronasal; o., orally; i.p., intraperitoneal; i.v., intravenous; end of study (P) planned, (E) death or euthanasia required.

## Data Availability

The data presented in the review are from sequences and papers published (and cited) in the References section, with abstracts accessible in PubMed.
